# Driving Factors of Geosmin Appearance in a Mediterranean River Basin: The Ter River Case

**DOI:** 10.3389/fmicb.2021.741750

**Published:** 2021-11-01

**Authors:** Carmen Espinosa, Meritxell Abril, Èlia Bretxa, Marta Jutglar, Sergio Ponsá, Núria Sellarès, Lídia Vendrell-Puigmitjà, Laia Llenas, Marc Ordeix, Lorenzo Proia

**Affiliations:** ^1^BETA Technological Center, University of Vic – Central University of Catalonia (UVic-UCC), Vic, Spain; ^2^CERM, Center for the Study of Mediterranean Rivers, University of Vic – Central University of Catalonia (UVic-UCC), Manlleu, Spain

**Keywords:** geosmin, field study, cyanobacteria, biofilm, global change, Mediterranean river

## Abstract

In recent decades, human activity coupled with climate change has led to a deterioration in the quality of surface freshwater. This has been related to an increase in the appearance of algal blooms, which can produce organic compounds that can be toxic or can affect the organoleptic characteristics of the water, such as its taste and odor. Among these latter compounds is geosmin, a metabolite produced by certain cyanobacteria that confers an earthy taste to water and which can be detected by humans at very low concentrations (nanogram per liter). The difficulty and cost of both monitoring the presence of this compound and its treatment is a problem for drinking water treatment companies, as the appearance of geosmin affects consumer confidence in the quality of the drinking water they supply. In this field study, the evaluation of four sampling sites with different physicochemical conditions located in the upper part of the Ter River basin, a Mediterranean river located in Catalonia (NE Spain), has been carried out, with the aim of identifying the main triggers of geosmin episodes. The results, obtained from 1 year of sampling, have made it possible to find out that: (i) land uses with a higher percentage of agricultural and industrial activity are related to high nutrient conditions in river water, (ii) these higher nutrient concentrations favor the development of benthic cyanobacteria, (iii) in late winter–early spring, when these cyanobacteria are subjected to both an imbalance of the dissolved inorganic nitrogen and soluble reactive phosphorus ratio, guided by a phosphorus concentration increase, and to cold–mild temperatures close to 10°C, they produce and release geosmin, and (iv) 1–2 weeks after cyanobacteria reach a high relative presence in the whole biofilm, an increase in geosmin concentration in water is observed, probably associated with the cyanobacteria detachment from cobbles and consequent cell lysis. These results could serve as a guide for drinking water treatment companies, indicating under what conditions they can expect the appearance of geosmin episodes and implement the appropriate treatment before it reaches consumers’ tap.

## Introduction

In the last decades, increasing pressures generated by human activities conjointly with global change trend led to worsened water quality in freshwater ecosystems, giving rise to several ecological issues. One of these problems, mainly caused by eutrophication and temperature rise, is the uncontrolled and unpredictable growth of algal blooms, frequently associated with organic compounds production, which can be toxic or can alter the water organoleptic characteristics, such as the taste and odor compounds (T&Os) (Clercin and Druschel, 2019). The presence of natural toxins in water often leads to bathing or consumption prohibition, whereas T&Os are a problem for drinking water treatment plants (DWTPs) due to the negative impact they have on the user’s perception of the drinking water quality (Ding et al., 2014).

Regarding human activities, their huge increase in the last century significantly affected rivers’ basin morphology and water quality (Rubio-Gracia et al., 2017). Particularly, basins’ land uses generally shifted from forestry-dominated to agricultural, livestock, and industrial. The increase of intensive agricultural activities in the catchments has been associated with higher nutrients and pesticides concentrations in river waters, which also receive a wide range of organic and inorganic chemical stressors, such as heavy metals, from industrial and urban areas (Drury et al., 2013; Argudo et al., 2020). The effects on water quality induced by the shift of land uses are acting conjointly with the alterations associated with climate change. Climatic conditions have changed in recent decades, being observed specifically in the Mediterranean regions, a clear decrease of the average rainfall, which is becoming much more sporadic and intense, and a relevant increase of the mean air temperature (Intergovernmental Panel on Climate Change [IPCC], 2019). These consequences of climate change take a greater relevance in rivers affected by water scarcity, such as those of the Mediterranean area, where there can be prolonged periods with a very low river flow, which can lead to a higher nutrients concentration, among other parameters (e.g., salts, heavy metals, and pesticides), due to the decreased dilution capacity of the system (Karaouzas et al., 2018). These conditions can trigger the appearance of algal blooms, with a large number of cases described worldwide (Clercin and Druschel, 2019; Ho et al., 2019), and that can finally lead to the appearance of T&O compounds (Watson et al., 2016).

Among the T&O compounds produced by microorganisms, geosmin has been described as the most common in freshwater ecosystems. This metabolite is mainly produced by certain cyanobacteria and actinomycetes, being the firsts associated with geosmin episodes in freshwaters, while actinomycetes usually have a terrestrial origen (Lukassen et al., 2019). Some of the main geosmin-producing cyanobacteria identified are *Oscillatoria* sp., *Dolichospermum* sp., *Lynghya* sp., and *Symploca* sp. (Smith et al., 2009). For a long time, due to the methodology of routine sampling, only the odorous potential of pelagic cyanobacterial taxa has been considered. However, recent studies suggested that most of the geosmin producers are benthic instead of pelagic cyanobacteria taxa (Jähnichen et al., 2011).

Most of the studies on drivers of geosmin appearance in the field have been carried out in reservoirs and lakes (Dzialowski et al., 2009; Harris et al., 2016), whereas only a few studies have investigated geosmin appearance in rivers and streams (Vilalta, 2004). Two of the main important factors associated with geosmin episodes in reservoirs have been described to be the excess of nutrient loads and alterations in its stoichiometric balance. In particular, it has been suggested that increasing nutrient concentrations and lower nitrogen to phosphorus ratio (TN:TP) can promote cyanobacteria growth and dominance in freshwater ecosystems (Olsen et al., 2016; Espinosa et al., 2021), giving rise to the appearance of geosmin episodes. Harris et al. (2016) found out that low TN:TP ratio conditions (<30:1 by mass) favor geosmin episodes in reservoirs, which could be related to an increase in cyanobacterial biovolumes at lower TN:TP ratios. In the Llobregat River (Catalonia, NE Spain), Vilalta (2004) found similar results, being geosmin concentration higher at TN:TP values close to 10:1, comparing with TN:TP = 94:1, when no geosmin was detected. From experiments carried out mainly under laboratory conditions, the appearance of geosmin has also been related to other factors such as light availability and temperature. Depending on the geosmin producers, the value of these factors differed, but in general, it has been described that low light availability together with low water temperatures favored intracellular geosmin formation (Zhang et al., 2009; Wang and Li, 2015; Alghanmi et al., 2018). Water flow also influence microbial production of geosmin, being its presence is higher under low water flow conditions (Jüttner and Watson, 2007; Espinosa et al., 2020).

As comment before, although there are studies on drivers of geosmin in reservoirs and lakes, very few have approached this topic in rivers. Geosmin occurrence can be a problem for the companies that exploit rivers to provide drinking water to the surrounding populations, as they do not know under which conditions is produced, and thus, they are unable to predict geosmin episodes. Small companies (water treated ≈ 1,500 L/day, drinking water users ≈ 22,000) cannot incorporate regular monitoring of geosmin concentrations in collected river waters, as analyses are complex and time-consuming, and they need specific equipment often not available in their labs. Moreover, the cost of the analytics can be high and make it difficult for the concerned companies to contract the geosmin analysis in external laboratories as a routine. The low capacity to predict geosmin presence in water leads to the reception of consumer complaints and economic losses associated with the decrease in water consumption supplied by the DWTPs. In that sense, there is a growing need to investigate and understand the drivers associated with the production of geosmin in rivers, helping DWTPs to be prepared for possible geosmin episodes and avoiding the possible costs associated with geosmin analysis *per se*.

In the Osona region (Catalonia, NE Spain), most of DWTPs collect water from the upper section of the Ter River to supply the nearby cities and villages (around 150.00 inhabitants). In recent years, they have suffered several geosmin episodes that have led to customers’ complaints due to the inability to applicate the required treatment on time. This situation has generated the need for a better understanding of the environmental factors associated with geosmin appearance. The main objective of this study was to determine the main triggers of geosmin episodes in the Ter River. To this aim, a 1-year field monitoring (2019) was carried out, analyzing a wide set of physicochemical and biological parameters at four sampling sites distributed along the upper part of the Ter River basin.

## Materials and Methods

### Study Site

**FIGURE 1:**
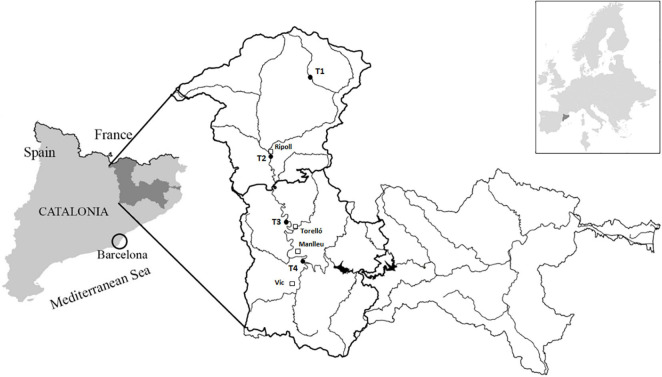
Ter River basin and sampling sites located in upper part (El Ripollès and Osona regions): T1 = Ter at Vilallonga de Ter municipality, T2 = Ter downstream Ripoll municipality, T3 = Ter at Colónia de Borgonyà (Sant Vicenç de Torelló municipality), and T4 = Ter at Gurb municipality.

The Ter River is located in the NE of Catalonia (Spain) (Figure 1). It is characterized by Pyrenean, pre-Pyrenean, and humid continental Mediterranean climate in the upper regions of its catchment (El Ripollès and Osona) and pre-coastal Mediterranean and coastal Mediterranean climates in the lower regions (Meteocat, 2019). The Ter River is affected by environmental fluctuations typical of the Mediterranean climate, with a higher probability of precipitation during spring and autumn and dry and warm summers. In the Ter River basin (208 km-long and 3,010 km^2^ of catchment area), several anthropogenic activities drastically affect water flow and quality. The most relevant impacts are: (i) the presence of small and frequent hydropower weirs, which significantly reduce river flow, (ii) livestock farming and intensive agriculture, leading to an increase of nutrients concentration in fields and in surface and groundwater, and (iii) a large reservoirs system, which supplies energy and raw water for drinking, agriculture, and industrial purposes to Barcelona city and Costa Brava area, which is dramatically affecting the river connectivity, and clearly dividing the catchment in two different areas: upstream and downstream reservoirs system. In this work, the upper part of the Ter River basin (upstream of the large reservoirs system) has been studied (Figure 1).

The river basin area evaluated in this study is included in the regions of El Ripollès and Osona, which cover a surface of around 2,000 km^2^. El Ripollès region is located at the head of the Ter River basin, starting in the Pyrenees, and it is characterized by coniferous forest, natural grasslands, broad-leaved forest, and moors and heathlands (CORINE Land Cover System, 2018). Located downstream, the Osona region is affected by strong anthropogenic pressures, being the main land uses related to non-irrigated arable fields, industrial and commercial units, and continuous and discontinuous urban fabrics (CORINE Land Cover System, 2018).

Four sampling sites (Figure 1) were chosen to perform a 1-year field study to identify the driving factors triggering geosmin episodes in the upper part of the Ter River basin. The most upstream sampling site (T1) was located 10.8 km downstream from the source of the Ter River in the Pyrenees, at Vilallonga de Ter municipality. T1 was considered as a reference sampling site, with the best water quality. The next sampling site was located along the Ter River, downstream the municipality of Ripoll (**T2**) (41 km downstream from the source). At this site, the Ter River has already received the input of the Freser tributary and some WWTPs effluents. The third sampling site was located at the Colonia de Borgonyà, few kilometers upstream of Torelló municipality (**T3**) and 66.3 km downstream from the source. This site was selected because it is one of the collection points of the drinking water company “Aigües d’Osona S.A.,” which supplies drinking water to Torelló municipality and surroundings. The last sampling site along the Ter River (**T4**) was located in Gurb municipality, 100 m upstream to the collection point of “Aigües de Vic S.A.,” the drinking water company that supplies the city of Vic and surroundings, and 81.8 km downstream Ter River source.

### Sampling Procedure and Physicochemical and Biological Analysis

During winter (January–March) and spring (April–June) seasons, weekly or biweekly field samplings were carried out, whereas from July to November (summer and autumn), monthly sampling campaigns were done. The higher sampling intensity during winter and spring was chosen because of the higher probability of geosmin occurrence during these seasons (Vilalta, 2004; Personal communication from drinking water companies). The nomenclature used in this study to identify the different sampling days includes the letter of the season (W = winter, Sp = spring, Su = summer, and A = autumn) followed by the number of the sampling day in this season, being, for example, W6, the name given to the sixth day of sampling in winter.

#### Water Samples

The following physicochemical parameters were measured *in situ* with specific probes: temperature, dissolved oxygen concentration, and oxygen saturation (YSI professional plus, YSI Incorporated, United States), pH (XS pH7+ DHS), and electrical conductivity (XS COND 7+). Water samples were taken and filtered through 0.2-μm nylon membranes filters (Merck Millipore) before the analysis of soluble reactive phosphorus (SRP), N-NH_4_^+^, N-NO_2_^–^, and N-NO_3_^–^. The volume filtered for SRP and N-NH_4_^+^ was 10 ml and for N-NO_2_^–^ and N-NO_3_^–^ was 50 ml, and the analyses were performed following the protocols established by Murphy and Riley (1962); Reardon et al. (1966), and Rand et al. (1976), respectively. The dissolved inorganic nitrogen (DIN):SRP ratio was calculated and determined as DIN divided by SRP in molar quantities. DIN concentration was determined as the sum of ammonium (N-NH_4_^+^), nitrite (N-NO_2_^–^), and nitrate (N-NO_3_^–^) concentrations. Furthermore, 1 L of water was taken for the analysis of turbidity, suspended solids, and organic matter. Water turbidity was measured using a turbidimeter (HI 98713, HANNA Instruments). The organic matter present in water samples was estimated from the absorbance values measured at 254 nm using a spectrophotometer (NanoPhotometer^TM^ P-360, INTEM), and the suspended solids were obtained following APHA (2005) and using a Forced air oven, MEMMERT IFE500. All samples were stored at –20°C until analysis.

A 1-L opaque glass bottle was used to collect the water sample for geosmin quantification. Bottles were stored at 4°C in dark conditions until analysis, which was performed within 48 h after collection to avoid degradation and volatilization. The protocol followed for geosmin analysis in water was described in Espinosa et al. (2021). Briefly, to analyze geosmin concentration, 50 ml of each water sample and 10 g of NaCl were added to a 100-ml opaque reaction vial and heated at 60°C for 25 min in agitation to favor geosmin volatilization. To extract the geosmin, a 65-μm polydimethylsiloxane/divinylbenzene fiber was used, and the separation and analysis of the extracted volatile compound were performed in a gas chromatography–mass spectrometry instrument (ISQ–TRACE GC ULTRA). The analytical detection limit was 2.5 ng/L, the analytical quantification limit was 8 ng/L, and the precision of the method was evaluated with the relative standard deviation (RSD ≤ 20%).

#### Biofilm Samples

Each sampling day, three cobbles were randomly taken from each sampling site to evaluate *in situ* the biofilm photosynthetic efficiency and the phototrophic community composition. The community photosynthetic efficiency (Y*eff*) and the minimum fluorescence yield (*F*_0_) (that can be used as an estimation of algal biomass) were measured with an amplitude modulated fluorimetry (Mini-PAM fluorometer Walz, Effeltrich, Germany), and the phototrophic community composition was evaluated with a BenthoTorch (bbe Moldaenke, Schwentinenta, DK). After that, each cobble was scrapped in 60 ml of water from the same sampling site to obtain a biofilm suspension. Aliquots of this suspension were used to analyze chlorophyll-*a* (Chl-*a*), performed as described by Jeffrey and Humphrey (1975), and ash-free dry mass as described in Espinosa et al. (2020). The Margalef index was also calculated as the quotient between the carotenoid/Chl-*a* ratio, being values obtained from the spectrophotometric reading of the sample at 430 nm (carotenoids and accessory pigments concentration) and 665 nm (Chl-a concentration), to obtain information about the maturity of the populations (Elosegui and Sabater, 2009). These samples were stored at –20°C until analysis.

### Data Treatment

The Kolmogorov–Smirnov test was performed to verify that the variables fulfilled the conditions of normal distribution, and if they did not, they were logarithmically transformed. Physicochemical and biological data were analyzed using an analysis of variance (ANOVA) using the “aov” function (“devtools” package) in RStudio software (version 3.6.0) being the sampling site, the season, and its interaction the factors evaluated. Significant results were tested *post hoc* with a Bonferroni test. Pearson correlation coefficient tests were carried out to explore the relationship between variables. Statistical significance was set at *p* < 0.05 for all tests performed. A redundancy discriminant analysis (RDA) was carried out to explore the potential relationship between the independent variables or potential drivers and the response variables (“vegan” and “tidyr” R packages). They were considered as potential drivers for all the water analytics except for geosmin concentration, which was considered as a response variable together with all the biofilm analytics. Forecasting models to predict the geosmin appearance according to the physicochemical data collected were generated by means of multiple regression analysis (MLR) and random forest (RF) models. The primary purpose of using a linear model was to provide a baseline against which to compare the non-linear RF model, an ensemble machine learning method that constructs a non-linear function based on an ensemble of simpler decision tree models (Kehoe et al., 2015). These models were tested for their ability to predict geosmin at different time delays, not just current conditions, to understand at what time scale geosmin can be predicted and which are the important predictors at each time lag. Models were calibrated for 11 different forecasting time lags ranging from the current level (i.e., no time lag, *t* = 0) up to 10 weeks in advance—in a 1-week increment. Linear (regression) and non-linear models (RF) were calibrated and validated (70–30%) on randomized subsets of the total dataset. The R function “lm” was used for the MLR, whereas RF models were developed with the “randomForest” package. The performance of the models was assessed by the multiple determination coefficient or *R*^2^ adjusted. The relative importance of the different predictors at different time lags was evaluated by using the “importance” function (“randomForest” package).

## Results

### Geosmin Concentration

**FIGURE 2:**
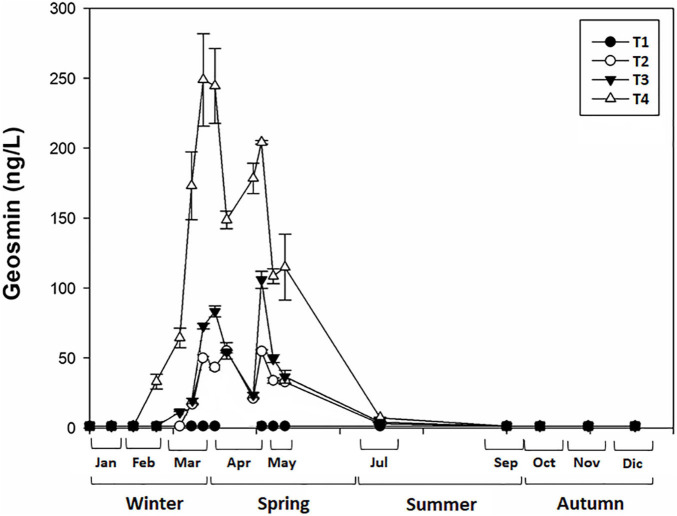
Mean value and standard deviation of geosmin concentration in water for different sampling sites (T1, T2, T3, and T4) in upper part of Ter River basin (NE Catalonia, Spain) at each sampling day.

The presence and concentration of geosmin in water varied throughout the year (2019), in which the present study was carried out (Figure 2). Statistically, geosmin presence in water was significantly affected by the sampling site (ANOVA, *F* = 10.378, *p* < 0.001), the season (*F* = 9.007, *p* < 0.001), and the interaction between site and season (*F* = 2.243, *p* < 0.05). Sampling site T4 was statistically different from the others (Bonferroni test: *p* < 0.001), being the one with the highest geosmin concentration, especially in spring (249 ± 33 ng/L) compared with the other seasons (*p* < 0.01).

### Physicochemical Parameters

Some of the evaluated physicochemical variables (Table 1) significantly changed depending on the sampling site, the season, and its interaction. The lowest values reported for most of the parameters evaluated were found at sampling site T1, mainly highlighting pH, electrical conductivity, temperature, and nutrient concentrations (ANOVA, *p* < 0.05). In contrast, sampling site T4 showed opposite results to T1, with very high values, especially for the nitrogen forms, whose concentration increased up to four times compared with T1, on average.

**TABLE 1:** Mean value and standard deviation of physicochemical variables evaluated for different sampling sites (T1–T4) in upper Ter River basin (NE Catalonia, Spain) in 2019: in winter (W), spring (Sp), summer (Su), and autumn (A).

		pH	EC (μS/cm)	Temp. (°C)	DO (mg/L)	Sat. (%)	N-NH_4_^+^ (μg/L)	N-NO_2_^–^ (μg/L)	N-NO_3_^–^ (mg/L)	P-PO_4_^3–^ (μg/L)	DIN:SRP	SS (mg/L)	Turbidity (NTU)	OM (A_254 nm_)
T1	W	7.8 ± 0.6	153 ± 32	4.6 ± 1.3	12.1 ± 1.0	99 ± 16	55 ± 40	2 ± 1	0.37 ± 0.07	66 ± 68	27 ± 19	29 ± 19	13 ± 11	0.40 ± 0.34
	Sp	7.9 ± 0.3	149 ± 37	7.4 ± 1.6	11.6 ± 1.0	105 ± 11	26 ± 13	3 ± 2	0.42 ± 0.06	96 ± 94	19 ± 14	8 ± 4	12 ± 10	0.23 ± 0.35
	Su	8.1 ± 0.4	150 ± 24	12.9 ± 3.0	10.1 ± 1.0	98 ± 10	45 ± 34	9 ± 10	0.30 ± 0.20	36 ± 50	31 ± 20	6 ± 5	10 ± 7	0.30 ± 0.39
	A	7.8 ± 0.4	148 ± 30	7.0 ± 2.9	11.9 ± 2.0	100 ± 18	21 ± 14	3 ± 5	0.33 ± 0.22	18 ± 10	60 ± 41	13 ± 14	10 ± 7	0.34 ± 0.44
T2	W	8.3 ± 0.4	250 ± 46	6.2 ± 2.1	12.7 ± 1.1	106 ± 10	79 ± 35	7 ± 5	0.61 ± 0.16	54 ± 40	42 ± 43	16 ± 11	23 ± 9	0.23 ± 0.26
	Sp	8.3 ± 0.4	291 ± 61	9.5 ± 1.2	11.6 ± 0.7	108 ± 8	54 ± 27	9 ± 8	0.61 ± 0.14	48 ± 25	37 ± 24	23 ± 14	20 ± 8	0.13 ± 0.15
	Su	8.2 ± 0.5	293 ± 67	16.0 ± 3.4	10.2 ± 1.6	104 ± 12	61 ± 33	20 ± 26	0.78 ± 0.54	29 ± 28	74 ± 44	14 ± 13	21 ± 8	0.12 ± 0.09
	A	8.2 ± 0.5	324 ± 67	7.8 ± 4.4	12.8 ± 1.8	109 ± 13	45 ± 29	14 ± 16	0.48 ± 0.14	34 ± 25	69 ± 55	20 ± 17	22 ± 27	0.28 ± 0.34
T3	W	8.4 ± 0.3	343 ± 87	7.5 ± 2.4	12.9 ± 1.1	108 ± 9	80 ± 74	8 ± 5	0.75 ± 0.19	73 ± 57	50 ± 52	27 ± 16	18 ± 13	0.41 ± 0.29
	Sp	8.4 ± 0.1	302 ± 85	12.0 ± 2.5	10.8 ± 1.2	103 ± 10	75 ± 70	10 ± 6	0.67 ± 0.29	73 ± 55	31 ± 18	29 ± 26	19 ± 15	0.33 ± 0.33
	Su	8.5 ± 0.2	338 ± 91	17.2 ± 5.7	11.2 ± 3.1	107 ± 8	78 ± 38	19 ± 23	0.77 ± 0.50	82 ± 100	39 ± 24	19 ± 9	12 ± 5	0.35 ± 0.33
	A	8.4 ± 0.2	366 ± 76	9.7 ± 5.4	12.7 ± 2.1	111 ± 12	92 ± 119	36 ± 74	0.73 ± 0.45	80 ± 113	71 ± 56	27 ± 21	12 ± 9	0.35 ± 0.35
T4	W	8.4 ± 0.3	388 ± 70	7.4 ± 2.4	12.3 ± 0.7	104 ± 8	119 ± 49	14 ± 10	1.48 ± 0.68	87 ± 87	75 ± 61	31 ± 20	21 ± 15	0.35 ± 0.20
	Sp	8.3 ± 0.2	326 ± 75	13.6 ± 1.7	10.4 ± 2.0	101 ± 13	122 ± 90	19 ± 9	1.15 ± 0.42	79 ± 39	54 ± 38	34 ± 29	26 ± 21	0.35 ± 0.30
	Su	8.7 ± 0.5	374 ± 66	21.2 ± 4.3	9.5 ± 1.4	106 ± 12	93 ± 33	26 ± 21	1.23 ± 0.88	102 ± 76	54 ± 53	40 ± 5	18 ± 7	0.19 ± 0.15
	A	8.4 ± 0.4	405 ± 90	9.7 ± 5.4	11.9 ± 3.0	108 ± 15	75 ± 67	17 ± 9	1.60 ± 0.92	57 ± 47	150 ± 129	19 ± 20	11 ± 9	0.26 ± 0.17

*OM, organic matter; SS, suspended solids.*

Seasonality also had an effect in some of the physicochemical parameters evaluated, being in autumn when lower pH values and dissolved oxygen were recorded, together with higher nitrites concentration (Bonferroni test, *p* < 0.01 in all cases). Phosphate concentration and turbidity presented the highest values in spring (*p* < 0.001), when, in contrast, the DIN:SRP ratio showed lower results compared with summer and autumn (*p* < 0.01).

Geosmin concentration for all sampling sites and seasons was positively correlated with pH, electrical conductivity, turbidity, and phosphate concentration (Pearson’s correlation, *p* < 0.01 all cases) and negatively correlated with the DIN:SRP ratio (Pearson’s correlation, *p* < 0.05).

Considering that the geosmin peak was measured at T4, a specific analysis was carried on the sub-dataset regarding this sampling site. The analysis of correlations revealed that geosmin concentration was positively correlated with phosphorus concentration (Pearson’s correlation: *r* = 0.789, *p* < 0.01) and had a negative correlation with the DIN:SRP ratio (*r* = –0.868, *p* < 0.01). During spring, when the highest geosmin peak was detected, the DIN:SRP ratio was significantly lower (47:1 ± 16:1) compared with the other seasons. On the contrary, autumn was the season showing the highest DIN:SRP ratio (221:1 ± 34:1), due to the important decrease of phosphorus concentration (19 ± 11 μg P-PO_4_^3–^ μg/L).

### Biological Parameters

Biological parameters data showed significant differences depending on the sampling site, the season, and the interaction between both factors (Table 2). Sampling site T4 showed the highest values of Chl-*a* concentration and cyanobacteria presence, being significantly different from the rest of the sampling sites (Bonferroni test, *p* < 0.01). Seasonality also affected the biological parameters evaluated. Phototrophic community presented higher biomass values (estimated as microgram Chl-*a* per square centimeters) in summer (*p* < 0.01), whereas the *F*_0_ value was higher in autumn (*p* < 0.05), and Chl-*a* concentration and diatoms relative abundance had higher values in winter (*p* < 0.01). Correlation analysis revealed that diatom abundance was negatively correlated with geosmin concentration (*r* = –0.28, *p* < 0.05), but no significant correlation was found between geosmin and cyanobacteria presence.

**TABLE 2:** Mean value and standard deviation of biological variables evaluated for different sampling sites (T1–T4) in upper Ter River basin (NE Catalonia, Spain) in 2019, in winter (W), spring (Sp), summer (Su), and autumn (A).

		*F*_0_	Y*eff*	Cyanobacteria (μg/cm^2^)	Green algae (μg/cm^2^)	Diatoms (μg/cm^2^)	Cyanobacteria (%)	Green algae (%)	Diatoms (%)	Chl-*a* (μg/cm^2^)	MI	AFDM (g/m^2^)
T1	W	113 ± 43	322 ± 92	0.16 ± 0.08	0.02 ± 0.05	3.67 ± 2.80	7.97.5	0.0 ± 0.0	89.912.0	3.9 ± 3.9	2.0 ± 0.2	44 ± 59
	Sp	93 ± 54	350 ± 63	0.18 ± 0.07	0.19 ± 0.14	1.09 ± 0.33	13.36.8	0.0 ± 0.0	73.912.0	1.0 ± 0.8	1.9 ± 0.1	74 ± 69
	Su	89 ± 30	368 ± 132	0.91 ± 1.03	0.00 ± 0.00	0.99 ± 0.39	38.225.7	0.0 ± 0.0	61.825.7	0.6	2.1	46 ± 61
	A	165	277	0.54 ± 0.61	0.03 ± 0.05	1.82 ± 0.30	19.818.8	0.0 ± 0.0	79.320.4	3.2 ± 1.8	2.0 ± 0.2	21 ± 18
T2	W	160 ± 117	489 ± 80	0.45 ± 0.33	0.01 ± 0.03	3.21 ± 2.42	11.45.9	0.0 ± 0.0	87.03.3	23.9 ± 21.7	2.2 ± 0.1	299 ± 271
	Sp	127 ± 58	435 ± 145	0.49 ± 0.27	0.45 ± 0.31	1.49 ± 0.62	19.55.9	0.0 ± 0.0	62.28.9	2.5 ± 1.6	2.0 ± 0.2	140 ± 111
	Su	133 ± 80	452 ± 278	1.02 ± 0.35	0.00 ± 0.00	1.74 ± 0.99	39.521.9	0.0 ± 0.0	60.521.9	11.1	2.5	17 ± 1
	A	227	630	0.42 ± 0.36	0.00 ± 0.00	2.32 ± 1.43	14.55.9	0.0 ± 0.0	85.55.9	7.0 ± 8.0	2.3 ± 0.1	48 ± 41
T3	W	189 ± 64	317 ± 106	0.78 ± 0.48	0.00 ± 0.00	3.59 ± 2.32	18.86.2	0.0 ± 0.0	81.26.2	18.0 ± 17.3	2.4 ± 0.1	134 ± 133
	Sp	149 ± 43	355 ± 134	0.58 ± 0.26	0.03 ± 0.04	1.74 ± 0.53	24.86.7	0.0 ± 0.0	74.06.4	7.2 ± 3.1	2.3 ± 0.1	196 ± 208
	Su	299 ± 108	501 ± 111	1.66 ± 1.12	0.11 ± 0.16	2.38 ± 0.34	37.715.2	0.0 ± 0.0	58.810.3	8.1	2.3	11.2 ± 1.9
	A	335	530	1.05 ± 0.25	0.00 ± 0.00	2.98 ± 0.71	26.24.3	0.0 ± 0.0	73.84.3	12.3 ± 2.7	2.3 ± 0.2	81 ± 74
T4	W	219 ± 115	473 ± 106	0.91 ± 0.63	0.00 ± 0.00	4.19 ± 3.35	21.313.7	0.0 ± 0.0	78.713.7	31.2 ± 25.4	2.2 ± 0.2	269 ± 288
	Sp	160 ± 50	467 ± 118	0.86 ± 0.64	0.02 ± 0.05	1.80 ± 0.41	28.911.1	0.0 ± 0.0	70.311.6	10.3 ± 6.2	1.8 ± 0.3	140 ± 102
	Su	206 ± 0	570 ± 48	1.26 ± 0.31	0.02 ± 0.03	1.83 ± 0.02	40.26.2	0.0 ± 0.0	59.05.1	11.0	3.0	7
	A	318	499	0.80 ± 0.68	0.00 ± 0.00	5.10 ± 2.41	15.114.8	0.0 ± 0.0	84.414.8	12.9 ± 4.4	2.4 ± 0.2	48 ± 22

*AFDM, ash-free dry mass; MI, Margalef index.*

Evaluating the sampling site with the highest geosmin concentration (T4), it was found that geosmin concentration was negatively correlated with the *F*_0_ (Pearson’s correlation: *r* = –0.681, *p* < 0.01), diatoms biomass (*r* = –0.661, *p* < 0.01), Margalef index (*r* = –0.643, *p* < 0.01), and Chl-*a* concentration (*r* = –0.533; *p* < 0.05).

### Geosmin Drivers

**FIGURE 3:**
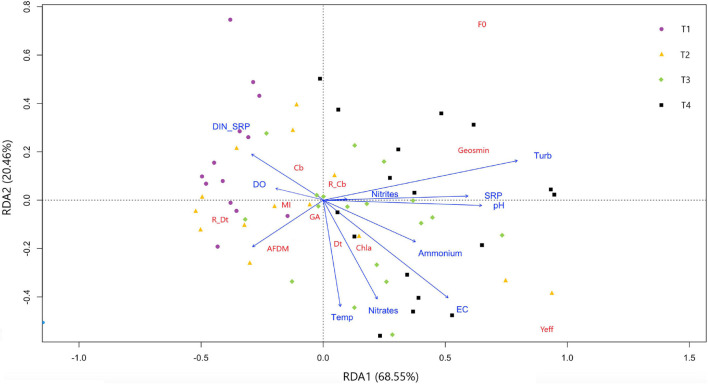
Redundancy discriminant analysis showing response parameters distribution based on drivers evaluated in four different sampling sites in upper Ter River basin (NE Catalonia, Spain) in 2019. Axes 1 and 2 combined explain 89% of variance. Drivers: pH, temperature (Temp), electrical conductivity (EC), dissolved oxygen (DO), turbidity, nitrite (N-NO_2_^–^), nitrate (N-NO_3_^–^), ammonium (N-NH_4_^+^), and phosphate concentration (P-PO_4_^–3^), and nitrogen to phosphorus ratio (DIN:SRP). Response variables: geosmin, minimum fluorescence yield (*F*_0_), photosynthetic efficiency (Y*eff*), chlorophyll-*a* concentration (Chl*a*), ash free dry mass (ADFM), Margalef index (MI), cyanobacteria (Cb), diatoms (Dt) and green algae (GA) biomass, and relative abundance of cyanobacteria (R_Cb) and diatoms (R_Dt).

An RDA was performed with the objective to identify the relationship between the potential drivers (all the physicochemical variables except geosmin) and the response variables (all the biological variables together with geosmin) measured at the different sampling sites (Figure 3). The independent variables in the first two RDA dimensions (RDA1 and RDA2) explained 89% of the total variance in the distribution of the response parameters.

This ordination clearly separates upstream (T1 and T2) from the downstream sites (T3 and T4) along the first axis, which explains the 68.8% of variability, and is mainly driven by increasing nutrient concentrations along the Ter River gradient. The DIN:SRP relationship also influences the distribution of the sampling sites, with T1 being the site with the lowest mean values. Geosmin concentration was mainly affected by the concentration of phosphates and nitrites, the turbidity, pH, and DIN:SRP ratio values, being in agreement with the Pearson correlations found (all of them positively correlated with geosmin concentration except DIN:SRP ratio). The presence of cyanobacteria was favored by lower values of DIN:SRP, in contrast to diatoms, which presented higher values under high nitrate concentration conditions. This last situation also favored high Chl-*a* values and biofilms with higher photosynthetic efficiency (Y*eff*).

**FIGURE 4:**
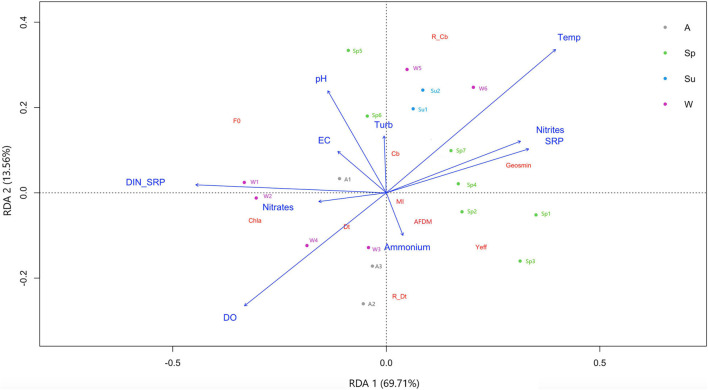
Redundancy discriminant analysis showing biological parameters distribution at T4 sampling site based on physicochemical variables evaluated in upper Ter River basin (NE Catalonia, Spain) in 2019. Axes 1 and 2 combined explain 83.3% of variance. Seasons are winter (W), spring (Sp), summer (Su), and autumn (Au). Drivers: pH, temperature (Temp), electrical conductivity (EC), dissolved oxygen (DO), turbidity, nitrite (N-NO_2_^–^), nitrate (N-NO_3_^–^), ammonium (N-NH_4_^+^), and phosphate concentration (P-PO_4_^–3^), and nitrogen to phosphorus ratio (DIN:SRP). Response variables: geosmin, minimum fluorescence yield (*F*_0_), photosynthetic efficiency (Y*eff*), chlorophyll-*a* concentration (Chl*a*), ash free dry mass (ADFM), Margalef index (MI), cyanobacteria (Cb), diatoms (Dt) and green algae (GA) biomass, and relative abundance of cyanobacteria (R_Cb) and diatoms (R_Dt).

Considering that the highest geosmin concentrations were found at the T4 sampling site, a specific RDA was performed for this sub-dataset (Figure 4). The potential drivers for RDA1 and RDA2 explain 83.3% of the total variance in the distribution of response parameters in T4. This analysis clearly shows the effect of seasonality in the appearance of geosmin at this sampling site. In particular, the first axis, which explains the 69.71% of the variability, separates winter and autumn from spring and summer samplings and is mainly driven by increasing phosphorus and nitrites concentrations and decreasing DIN:SRP values that again seem to be related to cyanobacteria abundance and geosmin concentration. More specifically, on the first sampling day of winter (W1), the tendency was to move from high DIN:SRP ratio values (187:1) toward lower values (48:1 in W6). This gradual decrease of DIN:SRP ratio was mainly guided by a relevant increase of phosphorus concentration (from 32 ± 3.5 to 70 – 100 ± 10.4 μg P-PO_4_^3–^/L) conjointly with a decrease in nitrate concentration (from 2.98 ± 0.02 to 1.26 ± 0.18 mg N-NO_3_^–^/L) and coincide with the first detection (W4) and gradual increase of geosmin concentration during late winter–early spring. During this period, water temperature increases from 4.2°C in W1 to 9.5°C in W6, keeping with average values of 13.6 ± 1.7°C in spring. The same physicochemical parameters related to geosmin concentration are those that seem to be linked to the cyanobacteria presence in the biofilm (both absolute concentration and relative abundance), with higher values at the end of winter and in summer. Conversely, the highest concentration of Chl-*a* and diatoms occur in autumn and early winter, driven by higher values of the DIN:SRP ratio, nitrates concentration, and dissolved oxygen.

An MLR and an RF model between the physicochemical variables (drivers) and geosmin concentration was performed at different time lags (weeks) to identify which factors are the best predictors of geosmin concentration.

**FIGURE 5:**
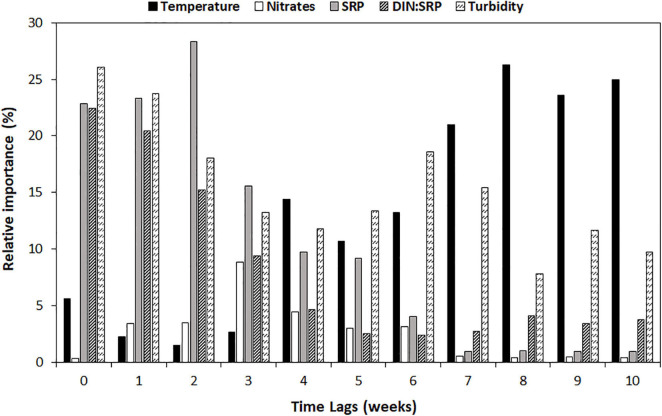
Relative importance (in %) of main predictors [temperature, in degrees Celsius; nitrates concentration, in N-NO_3_^–^ mg/L; phosphate concentration (SRP), in P-PO_4_^3–^ mg/L; DIN:SRP ratio, in mol:mol; and turbidity, in NTU] at different time lags (in weeks) in random forest model.

Both RF and MLR modeling techniques led to good model fits, but the RF was the better of the two at each time lag (based on *R*^2^ adjusted) (Table 3). At *t* = 0 weeks (w), the *R*^2^ adj. provided by the RF is 0.70, increasing up to 0.81 at *t* = 2w, and decreasing below 0.5 at 8 weeks. A similar pattern is found for the MLR, but in this case, the higher predictive accuracy is 0.62 (*t* = 2w) and decreases below 0.5 at 6 weeks. The analysis of relative predictor importance revealed patterns associated with the time lag of prediction (Figure 5 and Supplementary Figure 1). Before lag 4 weeks (*t* = 5–*t* = 10w), nitrates concentration and temperature are the parameters with higher relative importance within the RF model (≈10–20%). From that moment on, the phosphorus concentration, the DIN:SRP ratio, and the turbidity value begin to gain relevance, being at *t* = 2w when the phosphorus concentration reaches its maximum (28.3%). At this time, there is also a decrease in the relative importance of nitrate concentration, which coincides with an increase in the weight of the DIN:SRP ratio within the model, which is one of the main predictors of geosmin concentration among time-lags 0–2w (22.4–15.2%). At *t* = 0 and *t* = 1 week, turbidity is the parameter with the higher relative importance (26.1 and 23.8%, respectively).

**TABLE 3:** Predictive accuracy as measured by *R*^2^ adjusted for different time lags (in weeks), for multiple linear regression (MLR) and random forest (RF) models in upper Ter River basin (NE Catalonia, Spain) in 2019.

*R*^2^ adj.	Time lag (weeks)										
	0	1	2	3	4	5	6	7	8	9	10
MLR	0.61	0.58	0.62	0.55	0.49	0.50	0.47	0.45	0.36	0.35	0.30
RF	0.70	0.73	0.81	0.77	0.74	0.68	0.57	0.52	0.46	0.46	0.43

## Discussion

This study, carried out in the upper part of the Ter River basin, suggested the physicochemical parameters that could trigger geosmin appearance in a river where benthic cyanobacteria are the main geosmin producers.

### Geosmin Episodes in the Upper Ter River Basin

During the study, geosmin concentration varied depending on the sampling site and the season (Figure 2). Sampling sites differed mainly in the nutrient’s concentration, being higher in T3 and T4, located downstream. The nutrient concentration pattern measured along the upper river Ter could be explained by nearby land uses, which in the Osona region (where T3 and T4 are located) are predominantly related to agriculture (35% of the total area) and urban and industrial development (CORINE Land Cover System, 2018), whereas El Ripollès region, where T1 and T2 sampling sites were located, was characterized by a high percentage of forest and pastures (between 31.2 and 42.5%). The lower levels of point and non-point pollution sources, related to dominant land-use together with the river continuum concept, could help to explain the lower nutrient concentrations found at these sampling sites (Table 1). Furthermore, forests and pastures are environments that can contribute to reducing surface erosion and soil sediment runoff, which are among the main diffuse sources of phosphorus to freshwater rivers. Ongley et al. (2010) reported that agriculture contributes >50% of the total nutrient load, and the fraction of this total load is highly dependent on the proportion of agriculture in the watershed (Shi et al., 2017). Furthermore, agricultural activities are used to have a marked seasonality, with spring being the season that, due to a greater number of rains and higher temperatures, favors better growth and development of crops. This agrees with what is observed in the agricultural activity of Osona, where most of the crops are cereals (53.7%) and forages (43.1%), whose sowing time is in late winter and spring (mainly in March) (Departament d’Agricultura, Generalitat de Catalunya). This makes agricultural land uses have a greater impact on water quality in winter and spring, when planting occurs and crops are fertilized. Moreover, in spring, a larger amount of rainfall could cause fertilizer used in the crops to overflow into the river leaches, directly or through the contribution of groundwater, thereby leading to the deterioration of river water quality. Specifically, ammonium and phosphorus can be easily absorbed by soil particles, then being transported to streams and rivers in events of soil erosion and runoff (Withers and Jarvie, 2008). In the opposite way, nitrate is highly soluble and mobile, and when there is an excess of nitrates, it is leached to groundwater and reaches the rivers through underground flows (Grizzetti et al., 2011). The difference in the mobilization dynamic of nitrogen and phosphorus from the surface can give rise to changes in the DIN:SRP ratio.

Both the concentration of nutrients and the DIN:SRP ratio are factors related to the development of certain cyanobacteria, being described that high nutrient concentrations favor the appearance of cyanobacterial blooms (Dodds and Smith, 2016; Lee et al., 2017), in many cases related to geosmin production (Ding et al., 2014). Similar results were observed in this field study, where higher nutrient concentration may have generated favorable conditions for the cyanobacterial development within biofilm communities (0.91 ± 0.58 μg/cm^2^ in T4 compared with 0.33 ± 0.44 μg/cm^2^ in T1, mean annual value). Some studies have described that a high nitrogen concentration is necessary for cyanobacteria blooms to occur (>0.8 mgTN/L, Xu et al., 2015; ≈0.1 mgN-NH_4_^+^/L, ≈1.1 mgN-NO_3_^–^/L, Espinosa et al., 2021). Perkins et al. (2019) pointed out that the ammonium concentration was key for stimulating cyanobacteria development and production of T&O compounds, specifically revealing that metabolites were associated with high ammonium relative to nitrate. In the opposite way, a study performed by Harris et al. (2016) suggested that relatively low NO_3_:NH_3_ ratios provide conditions that favor the production of cyanobacterial metabolites. A previous study by Jankowiak et al. (2019) described that phosphorus availability excess may stimulate cyanobacteria blooms, and some studies have pointed out that total phosphorus (TP, both organic and inorganic forms) concentration had to be between 20 and 100 μg TP/L to control the growth of cyanobacteria (Sharma et al., 2011; Li et al., 2018). Moreover, Graham et al. (2004) identified the dominance of cyanobacteria often greatest when the total nitrogen (TN):TP ratio was low (<29:1 by mass), similar to the results found by Harris et al. (2016) in a study carried out in four North American reservoirs. Vilalta et al. (2003), in a study performed in the Llobregat River (Catalonia, NE Spain), suggested that an unbalanced proportion between nitrogen and phosphorus had an effect on benthic geosmin production, being its appearance favored under low TN:TP ratios (TN:TP = 10:1). The study carried out under laboratory conditions by Espinosa et al. (2021) showed that high nutrient concentration, together with a low DIN:SRP ratio (DIN:SRP = 4:1), triggered the production and release of geosmin by the *Oscillatoria* cyanobacterium present in the biofilm. This difference in the results could be due to factors related to the study system, such as stratification, redox potential, and resuspension of nutrients in reservoirs. However, it could be pointed out that a minimum of nitrogen should be necessary to favor cyanobacteria development. Nevertheless, to trigger geosmin production, an increase in the phosphorus concentration must occur, leading to an imbalance in the DIN:SRP ratio or TN:TP ratio. A similar situation has been observed in this study, as both cyanobacteria abundance and geosmin concentration was favored by high nutrient concentrations and lower DIN:SRP ratio, led by an increase of phosphorus concentration. The effect was clearly observed at T4, the sampling site with higher nutrient concentrations, where in late winter–early spring, there was a pronounced decrease of the DIN:SRP ratio value (Figure 4), associated with an important episode of geosmin. The interaction between DIN:SRP ratio and nutrient concentration seems to be an important driver favoring the production and release of this metabolite from benthic cyanobacteria in the Ter River.

### Mismatch Between Cyanobacteria Abundance and Geosmin Concentration

Although cyanobacteria presence and geosmin production seem to be favored by the same physicochemical factors, in this field study, no significant correlation was found between both parameters. This is somehow surprising, as these microorganisms are described as the main geosmin producers in freshwater ecosystems. One reason could be that an identification of the biofilm community at the genus level was not carried out in this study, and, as previously discussed, not all cyanobacteria are geosmin producers. On the other hand, it could be explained by the relative dynamics of geosmin production and release associated with the cyanobacteria life cycle. In fact, it has been described under controlled conditions that geosmin production mainly occurs during the growth phase, and its release to water is the direct consequence of biomass decomposition and/or cell lysis (Kim et al., 2018).

Although no biofilm community identification was carried out throughout the study, biofilm samples were taken from the T4 sampling site in late March for a parallel study carried out under laboratory conditions. These samples were characterized, and the cyanobacterium *Oscillatoria* sp. was identified as the main geosmin producer (Espinosa et al., 2021). Furthermore, a visual difference detected *in situ* was the presence of floating cyanobacterial mats coming from the biofilm in winter, whereas in summer, these mats were not observed (personal observation).

Regarding the mismatch found between cyanobacteria and geosmin in late winter–early spring, it could be explained by the cyanobacterial life cycle itself. Different studies performed by Hu et al. (2001, 2003) evaluating different cyanobacterial species explained that intracellular geosmin concentration increased in proportion to biomass. In addition to intracellular accumulation, it was observed that the concentration of geosmin in water began to increase. Once these cyanobacteria reached the stationary phase, there was a rapid decrease in intracellular concentration with a corresponding rapid increase of geosmin release, indicating that cell lysis and decomposition of geosmin producers may result in large spikes of these compounds in water supplies. Similar results were observed by Cai et al. (2017); Alghanmi et al. (2018), and Espinosa et al. (2021), supporting the idea that the majority of geosmin is normally retained with cyanobacterial cells during their growth, and release to the medium occur as a consequence of lysis and cellular decomposition. Different studies have pointed out that, depending on the cyanobacterial strain, the growth phase differs. Kruskopf and Du Plessis (2006) observed that *Oscillatoria simplicissima* reached the fast growth phase after 8 days, whereas Espinosa et al. (2021) found out the maximum *Oscillatoria* sp. presence at 16 days, and Jindal et al. (2011) described that *Oscillatoria formosa* could grow exponentially for 24 days before starting the stationary phase. In this field study, the relative abundance of cyanobacteria reached its peak after 1–2 weeks of gradual increase and 1 week later started to decrease, whereas geosmin in the water started reaching its peak 1 week later. This would confirm what was observed in several studies reporting geosmin release to water as a consequence of cyanobacterial biomass decomposition and/or cell lysis.

**FIGURE 6:**
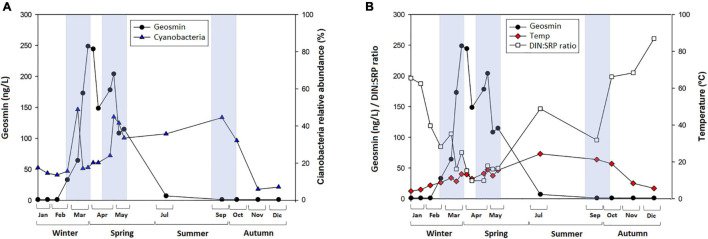
**(A)** Mean value and standard deviation of geosmin concentration (nanogram per liter), and cyanobacteria relative abundance (%) and **(B)** mean value and standard deviation of geosmin concentration (nanogram per liter) and DIN:SRP ratio (mol/mol), water temperature (°C) in T4 sampling site for different sampling in 2019, being Jan = January, Feb = February, Mar = March, Apr = April, May = May, Jul = July, Sep = September, Oct = October, Nov = November, and Dec = December. Blue boxes indicate time when cyanobacteria relative abundance in biofilm was high.

Despite the lack of correlation between cyanobacteria abundance and the geosmin concentration, the trends that have been observed, such as the ones shown in Figure 6, can help make a hypothesis about the geosmin drivers in the upper river Ter. This figure shows a notable increase in the cyanobacteria abundance during the first months of the year, reaching almost 50% of the biofilm community at the beginning of March and decreasing strongly 15 days later, coinciding with the geosmin peak (Figure 6A). Similar behavior was observed at the end of April. Nevertheless, this trend only occurs in winter and spring. In summer, cyanobacteria presence was also high, but geosmin was not detected, indicating that the factors favoring its production are not stable throughout the year and that a set of specific conditions have to co-occur to trigger geosmin production by benthic cyanobacteria. Some of the factors that differ between these two moments were the DIN:SRP ratio and the temperature, which presented significantly lower values in winter–spring than in summer (Figure 6B). The study performed by Alghanmi et al. (2018) pointed out that many cyanobacteria grow better under 25°C conditions, but this does not imply higher geosmin production. In fact, some studies have found higher geosmin concentration and production yield at 10°C compared with higher temperatures (25 and 35°C), indicating that lower temperatures could stimulate geosmin production and favor the accumulation of geosmin in cells (Zhang et al., 2009; Wang and Li, 2015). This would agree with our study, where higher geosmin concentration was observed at lower temperatures (close to 10°C), whereas at higher temperatures (20–25°C), geosmin levels were below the detection limit (Figure 6B). Moreover, considering that low light conditions have been described to favor intracellular geosmin production in river biofilms (Espinosa et al., 2020), the increased light availability occurring in summer may be an additional limiting factor for microbial geosmin production in the Ter River, as higher light incidence prevents gaseous vacuoles formation and geosmin production (Li et al., 2012). In fact, different studies have shown that at temperatures ≈20–25°C, higher light intensity hinders the production of geosmin by cyanobacteria (Oh et al., 2017; Alghanmi et al., 2018).

### Change in the Relative Predictor Importance

The change in the physicochemical conditions at the different sampling sites and seasons could explain the change in the relative importance of the parameters included in the models developed at different time lags. As shown in Figure 5, nitrate concentration and temperature are more relevant in predicting geosmin between lags 5 and 10 (1–2 and a half months in advance), which could indicate the need for high basal nitrate conditions (Xu et al., 2015; Espinosa et al., 2021). However, for a geosmin episode to occur, it seems that a gradual increase in the phosphorus concentration, whose maximum importance as a geosmin driver within the model is reached in lag 2-w, is needed to generate conditions that would favor the development of cyanobacteria biomass. The increase in phosphorus concentration generates an imbalance in the DIN:SRP ratio, whose values decrease as its relative importance in the model increases, up to its maximum at *t* = 0w (22.4%), indicating that this ratio must be kept low throughout the geosmin episode. These results agree with what was demonstrated by Espinosa et al. (2021) in a study performed under controlled conditions with biofilm communities collected from Ter River, in which cyanobacterial development (*Oscillatoria* sp.) and geosmin production were favored by higher nutrient concentration (both nitrogen and phosphorus) together with lower DIN:SRP ratio (4:1 compared with 64:1). Another parameter that has presented relatively high importance in the models is turbidity. This could have different explanations: the first one is that the lower incidence of light generated by greater turbidity promotes the development of low-light organisms, such as geosmin-producing cyanobacteria (Espinosa et al., 2020). In fact, other studies evaluating the light incidence with the Secchi Disk method have found a negative relationship between light penetration and geosmin concentration, supporting the idea that low light availability conditions may favor the development of geosmin-producing cyanobacteria (Dzialowski et al., 2009; Parr, 2014). Another explanation is that turbidity has been shown to be correlated with phosphorus concentration, mainly associated with the runoff process (Schilling et al., 2017), which has also been observed in this study. Finally, when the benthic geosmin–cyanobacteria producers are detached from the substrata, which coincide with the cells lysis and consequent geosmin release to the water column, it also releases different material, which can generate an increase in the turbidity value (i.e., solids trapped within thick biofilms in slow flow waters). This last point could explain why turbidity is the principal predictor at times 0 and 1.

The models developed with the database generated in the Ter River in 2019 have made it possible to know with a precision of 0.70–0.81 (considering 1 as the maximum possible value) the geosmin concentration up to a month–month and a half in advance. In addition, the results generated by the models indicate that the RF algorithm offers a great option for the evaluation of long-term ecological data sets. This model has also made it possible to identify the related parameters in each lag and the necessary changes of physicochemical parameters that should occur to increase the possibility of a geosmin episode being triggered. Knowing the relative importance of geosmin drivers, as evidenced by this study, drinking water treatment companies have the possibility of advancing to geosmin episodes based on the monitoring of easier and cheaper variables. In this way, they can have enough time to implement the required treatment and prevent geosmin from reaching the consumer’s tap, avoiding complaints from users by being able to offer quality drinking water continuously.

## Conclusion

Overall, this field study showed that factors directly and indirectly related to both global change and anthropogenic factors could be potential drivers of geosmin occurrence in Mediterranean rivers.

River stretches, which land uses of the surrounding areas favoring higher nutrient concentrations, are more susceptible to be affected by cyanobacterial blooms and geosmin episodes. For example, industrial and agricultural watersheds could lead to higher nutrient concentrations in river waters, which can favor certain cyanobacteria development (such as *Oscillatoria* sp.). Furthermore, agricultural watersheds are used to experience an increase of phosphorous concentration associated with planting and fertilization periods that may generate a DIN:SRP ratio decrease especially during late winter–early spring. This situation can favor that certain cyanobacterium would start to produce geosmin, which would be released into the water between 7 and 15 days after the cyanobacteria peak in biofilms, associated with the organism degradation or cell lysis.

These results could help to drinking water companies in the forecasting and management of geosmin episodes, being able to understand which ecological conditions are more prompt to favor the appearance of geosmin in the water collected from surface waters and thus allowing them to implement more targeted treatment regimens before geosmin reach the consumer’s tap.

## Data Availability Statement

The raw data supporting the conclusions of this article will be made available by the authors, without undue reservation.

## Author Contributions

All authors made substantial contributions to the conception or design of the work, or the acquisition, analysis, or interpretation of data for the work.

## Conflict of Interest

The authors declare that this study received funding from Aigües de Vic S.A. and Aigües d’Osona S.A. The funder was not involved in the study design, collection, analysis, interpretation of data, the writting of this article, or the decision to submit it for publication. The handling editor SM declared a past co-authorship with several of the authors CE, MA, SP, LV-P, LL, LP.

## Publisher’s Note

All claims expressed in this article are solely those of the authors and do not necessarily represent those of their affiliated organizations, or those of the publisher, the editors and the reviewers. Any product that may be evaluated in this article, or claim that may be made by its manufacturer, is not guaranteed or endorsed by the publisher.
