# Supplementary material for: Driving Factors of Geosmin Appearance in a Mediterranean River Basin: The Ter River Case
Source: Front Microbiol. 2021 Nov 1;12:741750. doi: 10.3389/fmicb.2021.741750 (PMC8591308; doi:10.3389/fmicb.2021.741750)
Supplement: Supplementary file 1 [file Data_Sheet_1.PDF]

frontiers

Driving factors of geosmin appearance in a Mediterranean river basin: the Ter river case

Carmen Espinosa[1,2], Meritxell Abril[1], Èlia Bretxa[2], Marta Jutglar[2], Sergio Ponsá[1], Núria Sellarès[2], Lídia Vendrell – Puigmitjà[1], Laia Llenas[1], Marc Ordeix[2] and Lorenzo Proia[1*]

[1]BETA Technological Center, University of Vic – Central University of Catalonia (UVic – UCC), Vic, Spain

[2]CERM, Center for the Study of Mediterranean Rivers, University of Vic – Central University of Catalonia (UVic-UCC), Manlleu, Spain

*** Correspondence:**
Lorenzo Proia
lorenzo.proia@uvic.cat

Figure 1. Relative importance (in %) of different predictors at different time lags (in weeks) in the random forest model: pH, Electrical conductivity (EC), water temperature (Temp.), dissolved oxygen (DO), ammonium concentration (Ammonium), nitrites concentration (Nitrites), nitrates concentration (Nitrates), phosphorus concentration (SRP), DIN:SRP ratio and turbidity (Turb.).
