# Supplementary figures and images for: Driving Factors of Geosmin Appearance in a Mediterranean River Basin: The Ter River Case
Source: Front Microbiol. 2021 Nov 1;12:741750. doi: 10.3389/fmicb.2021.741750 (PMC8591308; doi:10.3389/fmicb.2021.741750)

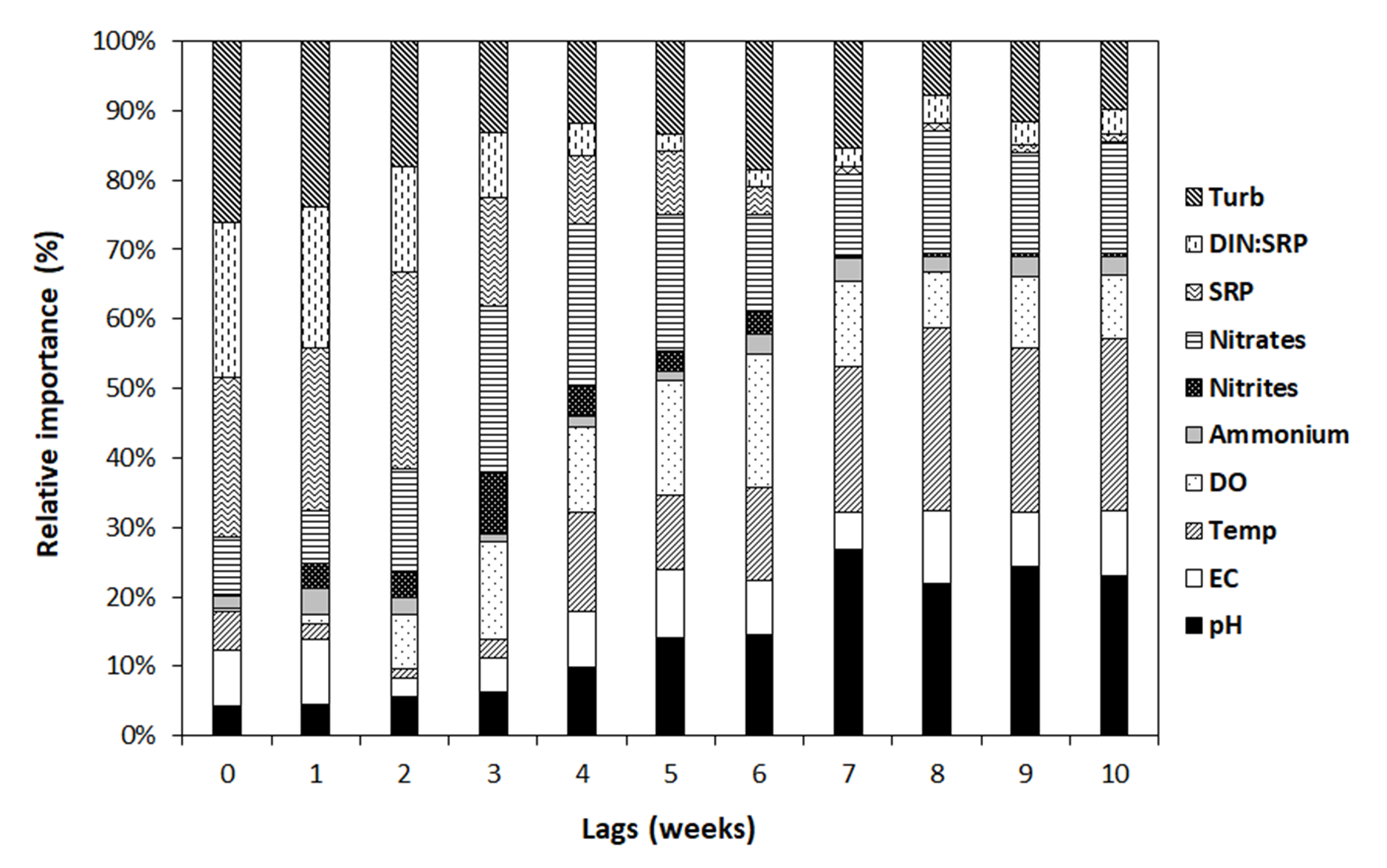

Supplement: Supplementary file 2 [file Image_1.TIF]
